# Tropical–temperate comparisons in insect seed predation vary between study levels and years

**DOI:** 10.1002/ece3.9256

**Published:** 2022-09-19

**Authors:** Wenlan Wu, Xiaoxue Wang, Tao Zhao, Wenfu Zhang, Shuai Fang, Yu Xu, Kai Zhang

**Affiliations:** ^1^ School of Life Sciences Guizhou Normal University Guiyang China; ^2^ Xishuangbanna Tropical Botanical Garden Chinese Academy of Sciences Mengla China; ^3^ Institute of Applied Ecology Chinese Academy of Sciences Shenyang China

**Keywords:** biodiversity, biotic interaction hypothesis, granivory, latitude, mast seeding, seed predation, seed predator satiation hypothesis, tropical versus temperate comparison

## Abstract

The biotic interaction hypothesis, which states the species interaction becomes stronger in the tropics, is deeply rooted in classic ecological literature and widely accepted to contribute to the latitudinal gradients of biodiversity. Tests in latitudinal insect–plant interaction have emphasized leaf‐eating insects on a single or a few plant species rather than within an entire community and mixed accumulating evidence, leaving the biotic interaction hypothesis disputed. We aimed to test the hypothesis by quantifying insect seed predation in a pair of tropical and temperate forest communities with similar elevations. We applied a consistent study design to sample predispersal seeds with systematically set seed traps in 2019–2020 and examined internally feeding insects. The intensity of seed predation was measured and further applied to tropical versus temperate comparison at two levels (cross‐species and community‐wide). Our results showed every latitudinal pattern associated with different study levels and years, that is, negative (greater granivory in the tropics in community‐wide comparison in 2020), positive (less granivory in the tropics in community‐wide and cross‐species comparison in 2019), and missing (similar level of granivory in the tropics in cross‐species comparisons in 2020). The cross‐species level analyses ignore differences among species in seed production and weaken or even lose the latitudinal trend detected by community‐wide comparisons. The between‐year discrepancy in tropical–temperate comparisons relates to the highly variable annual seed composition in the temperate forest due to mast seeding of dominant species. Our study highlights that long‐term community‐level researches across biomes are essential to assess the latitudinal biotic interaction hypothesis.

## Graphical abstract

The species interaction has been proposed to become intense towards the tropics. Here we compared insect seed predation between tropical and temperate forests and showed every latitudinal pattern associated with study levels and years.

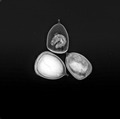

## 1 INTRODUCTION

Biodiversity increasing from the poles toward the equator is one of the most studied patterns in ecology (Gaston, 2000; Hillebrand, 2004; Mittelbach et al., 2007; Pontarp et al., 2019; Schemske et al., 2009). The biotic interaction hypothesis, which suggests the species interaction becomes stronger in the tropics (Darwin, 1859; Dobzhansky, 1950; MacArthur, 1972; Wallace, 1878), is widely accepted to contribute to the latitudinal gradients of biodiversity (Pontarp et al., 2019; Schemske et al., 2009; Wright, 2002), by ameliorating competitive exclusion via species‐specific (negative density‐dependent effect; Janzen, 1970, Connell, 1971) or ‐generalized (Paine, 1966) natural enemies, and/or by elevating speciation rate via evolutionary arms races (Coley & Kursar, 2014; Pontarp et al., 2019).

Latitudinal biotic interaction hypothesis draws much research interest, but the relationship between latitude and the importance of biotic interaction remains extensively disputed (Anstett et al., 2016; Coley & Aide, 1991; Coley & Barone, 1996; Comita, 2017; Freeman et al., 2020; Moles et al., 2011; Moles & Ollerton, 2016; Schemske et al., 2009; Zvereva & Kozlov, 2021). Until recently, most empirical studies have emphasized leaf‐eating insects on a single or a few plant species (e.g., Salazar & Marquis, 2012; Więski & Pennings, 2014), which were plausible to observe or manipulate, and provide positive, negative, or mixed results (Anstett et al., 2016; Zvereva, Zverev, Usoltsev, & Kozlov, 2020b). The species‐specific traits related to nutrition and defense may partially explain the variance. For example, higher levels of leaf herbivory and predispersal seed predation of an oak species at lower latitudes were probably due to lower plant defense and nutrition levels therein (Moreira et al., 2018, 2021). But such a pattern has yet to apply even to other oak species because global oak leaf defenses tend to become higher at lower latitudes (Pearse & Hipp, 2012). Meta‐analyses further found no significant latitudinal gradient in (pre‐)dispersal seed predation rates (Moles & Westoby, 2003; Zvereva & Kozlov, 2021).

Expanding the research scope to include more species in the community addresses some limitations of single‐species studies by increasing taxonomic replication (Anstett et al., 2016). Still, it faces challenges in choosing representative and comparable species sets, thus failing to reach a consensus about the biotic interaction hypothesis (Boyer, 2019; Chen et al., 2017). Furthermore, plant species play different roles in community structure and functioning, and it is questionable to assume that data averaged across selected species can reflect the community‐wide spatial pattern (Mottl et al., 2020; Zvereva, Zverev, Usoltsev, & Kozlov, 2020b). The solution is to re‐focus on the community level where the hypothesis was initially formulated (Coley & Aide, 1991; Coley & Barone, 1996): the percent leaf area of all plant species damaged by insects per year was expected to be greater in tropical forests as a whole, compared to temperate forests. However, the community‐wide level tests have been rare, even in insect folivory (Adams et al., 2011; Rheubottom et al., 2019; Zhang et al., 2011; Zvereva, Zverev, Usoltsev, & Kozlov, 2020b), let alone other types of species interaction.

Compared to leaf herbivory, predispersal seed predation by insects represents a unique but overlooked biotic interaction system (Gripenberg et al., 2019; Janzen, 1971; Lewis & Gripenberg, 2008; Xiao et al., 2017). The predispersal seed predators attack seeds developing on the tree, including members of Lepidoptera, Coleoptera, Diptera, and Hymenoptera (Sallabanks & Courtney, 1992). The insect seed‐eaters are engaged in close interactions with host plants and show high specificity (Novotny & Basset, 2005). Insect seed predators have essential ecological functions in inducing host plant fecundity loss and recruitment failure and regulating community composition and local species richness (Gripenberg et al., 2019; Weisser & Siemann, 2008). Furthermore, endophytic insect feeders are directly connected to seed mortality (Lewis & Gripenberg, 2008) and thus reflect the cost of seed predation to the host plants. However, meta‐analyses detected absent latitudinal trends in plant and predispersal seed predator interaction (Moles & Westoby, 2003, Zvereva & Kozlov, 2021), warranting further comparison of tropical and temperate seed predation intensity by insects.

We therefore studied seed predation by insects in a pair of tropical and temperate forest communities with similar elevations to test the biotic interaction hypothesis. To our knowledge, this is the first community‐level study to compare granivory across biomes. We specifically asked two questions: (1) Is seed predation intensity higher in tropical forests than in temperate forests? (2) Do the study levels (cross‐species vs. community‐wide level) influence the results of tropical versus temperate comparisons?

## 2 MATERIAL AND METHODS

### 2.1 Study sites

We conducted our study in two forest dynamics plots engaged in the Chinese Forest Biodiversity Network (CForBio, Feng et al., 2016) and Forest Global Earth Observatory network (CTFS‐ForestGEO, Anderson‐Teixeira et al., 2015). Both sites are in protected areas. The tropical forest plot (XSBN) is located in Xishuangbanna, Yunnan province (21.61°N, 101.57°E), with an area of 20 hm^2^. The elevation ranges from 709 to 869 m, the annual mean temperature is 21.8°C, and the mean annual rainfall is 1493 mm (Lan et al., 2008). The primary vegetation type is tropical seasonal rainforest, characterized by *Parashorea chinensis* in the canopy, *Sloanea tomentosa*, *Pometia pinnata*, *Pittosporopsis kerrii*, *Garcinia cowa*, and *Orophea laui* in the understory, and *Castanopsis echinocarpa* near the ridge (Dou et al., 2018). The temperate forest plot (CBS) is located in Mt. Changbaishan, Jilin Province (42.38°N, 128.08°E), with an area of 25 hm^2^. The elevation ranges from 791 to 809 m, the annual mean temperature is 3.6°C, and the mean annual rainfall is 700 mm (Zhang et al., 2008). The primary vegetation type is temperate coniferous and broad‐leaved mixed forest, characterized by *Tilia amurensis*, *Pinus koraiensis*, *Quercus mongolica*, and *Fraxinus mandshurica* (Qian et al., 2019).

### 2.2 Seed sampling

Seeds were sampled using 150 seed traps with a surface area of ~0.5 m^2^ erected in both forest plots following the Barro Colorado Island (BCI) forest dynamic plot (Wright et al., 2005). The seed traps were set systematically and as evenly as possible to ensure community‐wide sampling. Each plot was first divided into many 20‐ × 20‐m grids and then separated into four equal subplots by a cross band. The seed traps were finally systematically placed at the grids in the cross band and subplots (Dou et al., 2018; Zhang et al., 2008). The minimum distances between traps were 37 and 20 m for CBS and XSBN, respectively. The distribution maps of seed traps were shown in Dou et al. (2018) for XSBN and Zhang et al. (2008) for CBS.

All seeds and fruits falling into the traps were collected, sorted, and identified to species every 14–15 days. Seeds/fruits collected on the same day were hereafter referred to as a *batch* of samples. Seeds/fruits from each trap belonging to one species were then packed, counted, dried, and weighted. We surveyed seeds/fruits across the fruiting seasons of 2019 and 2020, that is, the whole year in tropical XSBN and around the Autumn season (August to November 2019, July to December 2020) in temperate CBS. A total of 26 and 27 batches of samples were collected in XSBN in 2019 and 2020, respectively. A total of seven and 13 batches of samples were collected in CBS in 2019 and 2020, respectively.

### 2.3 Seed predation by insects

**FIGURE 1:**
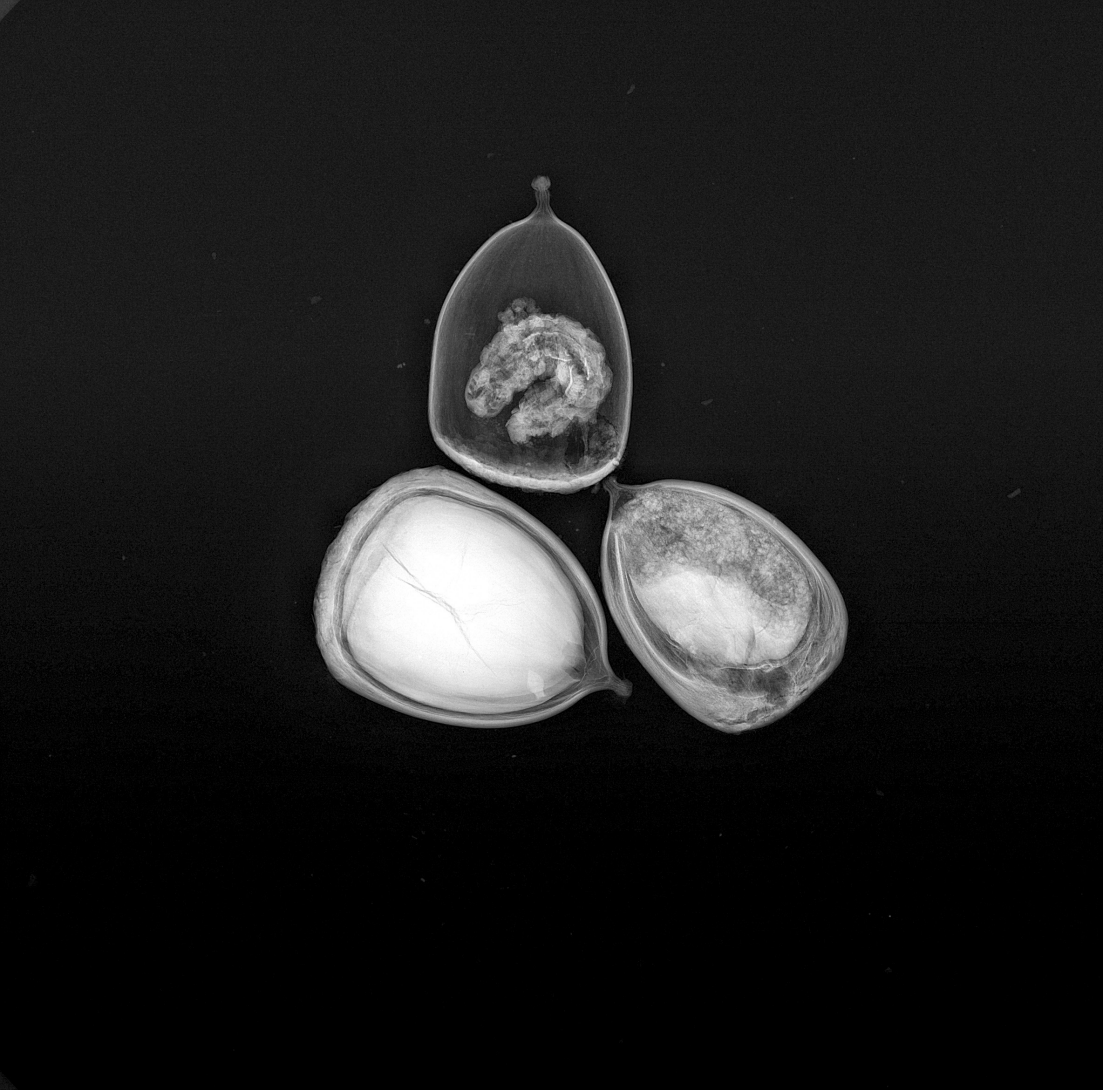
The radiograph of *Castanopsis megaphylla* collected from tropical XSBN in 2020. One intact (bottom left) and two damaged seeds, where a larva (top) and frass (bottom right) were detected, are shown here. The nuts are ~1 cm in length.

We examined all the seeds/fruits through an X‐ray machine (Faxitron X‐ray Corporation MX‐20‐DC12, Figure 1) and dissected the suspected seeds under a stereo microscope (OLYMPUS SZ61) to confirm the predation status. Seeds were considered to have been depredated by insects if there were (1) insects (including eggs/larvae/adults) or (2) frass, feeding damage, or entry/exit holes (Chen et al., 2017; Gripenberg et al., 2019; Jeffs et al., 2018). The intensity of seed predation may be derived with different approaches (Basset et al., 2018; Gripenberg et al., 2019; Jeffs et al., 2018), based on either both criteria (1, 2) to reflect the percentage of seed attacked by insects or only the criterion (1) to estimate the potential load of insects on seed mortality. Here, we referred to the former as *seed predation rate* (proportion of seeds showing signs of seed predator attack) and the latter as *incidence of seed predators* (proportion of seeds detected with insect predators). We weighted the two metrics by the average seed mass per trap × batch to account for the intraspecific variation in seeds for further analyses. We also retained unweighted metrics to facilitate comparisons with other studies. The incidence of seed predators was calculated for both 2019 and 2020 samples, and the seed predation rate was calculated for the 2020 sample only.

Regarding fruits containing multiple seeds, insect predation was analyzed for each seed separately. The survival of individual seed was our concern in analyses of predispersal seed predation (Chen et al., 2017). On minimal occasions, insect damage was restricted in the flesh and impeded an accurate estimate of seed predation intensity. We counted such a case as one seed predation event, which was unlikely to affect the general results due to the rarity of such cases. Immature seeds were included to minimize the errors in estimating seed predation intensity because premature abscission might be due to insect infection (Jeffs et al., 2018), and insect predation was indeed detected from immature seeds in our samples.

Tiny (seed length < 0.2 cm, Dou et al., 2018) and filamentous seeds, which were uncountable or undetectable for insect predation, were removed, including those from *Populus cathayana*, *P. koreana*, *P. simonii* var. *przewalski*, and *Betus platyphylla* in CBS (accounting for 0% and 2.90% of total seed mass in 2019 and 2002, respectively), and *Duabanga grandiflora*, *Ficus* spp., *Neolamarckia cadamba*, *Radermachera microcalyx*, *Terminalia myriocarpa*, *Uncaria macrophylla*, *Vernonia parishii*, and *V. sylvatica* in XSBN (accounting for 12.19% and 15.76% of total seed mass in 2019 and 2002, respectively). We acknowledged the removal of figs *Ficus* spp. could potentially bias the results of XSBN.

### 2.4 Simulated historical seed predation rate of *Pinus koraiensi* in temperate CBS

The dominant species of Korean pine (*Pinus koraiensis*) in CBS produced seeds in late September and early October, and seed production showed periodic dynamics every 2–3 years (Ji et al., 2002). After mast seeding in 2018, *P. koraiensis* produced almost no seeds in 2019 and just a few seeds in 2020. Rodents, birds, and humans further removed some seeds from the trees (Ji et al., 2002; Zhang et al., 2008), and only two seed cones were sampled in 2020. *P. koraiensis* was underrepresented in our samples, and we used historical data on seed attacks (Heilongjiang Fenglin Nature Reserve, 1975; Mao et al., 2003; Zhang et al., 2020) to simulate the seed predation rate of *P. koraiensis* by insects before the 1990s when humans began to collect seed cones for sale.

The primary insect predators of *P. koraiensis* seeds in Mt. Changbaishan were the moth *Dioryctria* spp. (Lepidoptera, Pyralidae), and the percentages of seed cone predated fluctuated annually from being very low (≤10%) to very high (50%–70%; Zhang et al., 2020), accounting for up to 19.7% of the seed predation rate (an average of 8.1 larvae and 115 seeds per cone, and four seeds predated per larva, 8.1 × 4 ÷ 115 × 70% = 19.7%, Heilongjiang Fenglin Nature Reserve, 1975). Note the simulated seed predation rate of *P. koraiensi* was an overestimation.

### 2.5 Statistical analysis

Analyses were performed using R 4.0.5 (R Core Team, 2021) and packages “nlme” 3.1–152 (Pinheiro et al., 2021), “caper” 1.0.1 (David et al., 2018), and “V.PhyloMaker” 0.1.0 (Jin & Qian, 2019).

We compared seed predation intensity between tropical and temperate forests at community‐wide and cross‐species levels. Community‐wide seed predation intensity was defined as the percentage of seed mass of all plant species predated by insects at a given site in a specified period, following Zvereva, Zverev, Usoltsev, and Kozlov (2020b). It was calculated for each batch of samples from both study sites. To test the study sites' effect on ln(1 + √x)‐transformed seed predation intensity, we implemented a linear model using generalized least squares (*gls* function in *nlme*). The errors allowed to be correlated and/or have unequal variances to address temporal autocorrelation and heterogeneity of variance. The batches were coded as the days since 1 January of the year and were included as a covariate. The correlation structure was specified as an autoregressive process of order 1 (*correlation* = corAR1), indicating the correlation between observations separated by a one‐time unit (i.e., 14–15 days) is likely to be more similar than those separated by larger time units (Zuur et al., 2009).

In cross‐species levels analyses, we applied the phylogenetic generalized least squares regression (*pgls* function in *caper*) to explore the study sites' effect on seed predation intensity while controlling for potential plant phylogenetic nonindependence (Mundry, 2014), which might play a key role in shaping insect herbivory assembly (Turcotte et al., 2014; Weiblen et al., 2006). The phylogenetic signal (measured as λ, lambda = ‘ML’) was adopted to reflect the extent to which seed predation intensity was statistically related to plant phylogeny (Symonds & Blomberg, 2014). The response in the model was the ln(1 + √x)‐transformed seed predation intensity of each species, pooled across all traps × batches per year. The fruit types (fleshy fruit and dry fruit, Yang et al., 2010) and seed mass were possibly confounding variables (Basset et al., 2018; Chen et al., 2017; Gripenberg et al., 2019) and were thus included as covariates. The seeds of gymnosperms, including *Pinus koraiensis*, *Gnetum pendulum*, *G. montanum*, and *Abies holophylla*, were surrounded by no protective tissue like flesh against insect predators, thus were functionally classified as dry fruits. Only species with ≥50 seeds were retained in the analyses. The plant phylogeny was constructed with taxonomies against a mega tree as our framework (*phylo.maker* function in *V. PhyloMaker*). The plant taxonomy followed *Flora of China* (Editorial Committee of Flora of China, 1989–2013).

## 3 RESULTS

In the tropical forest plot (XSBN), 15,128 seeds (dry weight: 3958.59 g) from 154 species in 127 genera and 60 families were collected in 2019 (Table 1). The top plant species contributing to seed production was *Castanopsis echinocarpa*, yielding 14.80% of seed mass (19.43% of seeds). The annual incidence of seed predator was 8.40% (Table 1). A total of 23,589 seeds (dry weight: 4346.75 g) from 159 species in 129 genera and 61 families were collected in 2020. The top dominant plant species contributing to seed production was *Pometia pinnata*, yielding 11.67% of seed mass (2.79% of seeds). The annual seed predation rate and incidence of seed predator were 24.73% and 6.72%, respectively (Table 1).

**TABLE 1:** Seed samples and seed predation intensity in two study sites in 2019–2020

Site	Parameters	2019		2020		
		Seed number (seed mass)	Incidence of seed predators	Seed number (seed mass)	Seed predation rate	Incidence of seed predators
Tropical XSBN	Annual measurements	15,128	8.40%	23,589	24.73%	6.72%
		(3958.59 g)		(4346.75 g)		
	Community‐wide measurements (per batch)	‐	9.73 ± 1.72%	‐	24.66 ± 1.62%	6.78 ± 0.86%
	Cross‐species measurements (per species)	‐	7.64 ± 1.46%	‐	19.37 ± 2.23%	5.11 ± 0.79%
Temperate CBS	Annual measurements	15,614	17.93%	148,586	5.23%	2.00%
		(1093.50 g)		(3262.18 g)		
	Community‐wide measurements (per batch)	‐	17.56 ± 1.07%	‐	5.73 ± 1.02%	1.84 ± 0.43%
					[8.10 ± 1.73%]	
	Cross‐species measurements (per species)	‐	9.42 ± 3.03%	‐	7.01 ± 1.84%	2.42 ± 0.79%
					[8.28 ± 2.08%]	

*Note*: Measurements incorporating simulated data of *Pinus koraiensis* are shown in square brackets. Mean ± SE values are presented. Cross‐species measurements unweighted by seed mass, which ignore intraspecific variations in the seed mass, are shown in Table A1.

In the temperate forest plot (CBS), 15,614 seeds (dry weight 1093.50 g) from 10 species in four genera and four families were collected in 2019. 79.34% of seeds came from *Fraxinus mandshurica*, accounting for 52.36% of total seed mass. The annual incidence of seed predator was 17.93% (Table 1). A total of 148,586 seeds (dry weight 3262.18 g) from 14 species in eight genera and eight families were collected in 2020. 91.38% of seeds come from *Tilia amurensis*, accounting for 74.50% of seed mass. The annual seed predation rate and incidence of seed predator were 5.23% and 2%, respectively (Table 1).

**FIGURE 2:**
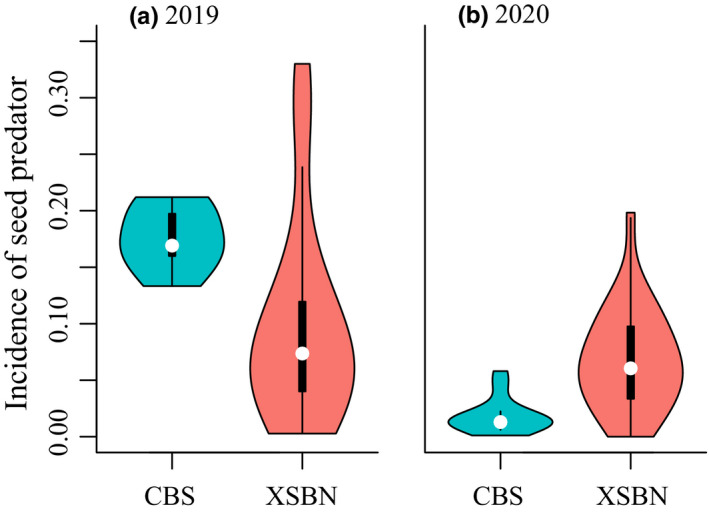
The tropical–temperate comparisons of the incidence of seed predators at the community‐wide level in (a) 2019 and (b) 2020. Opposite patterns are shown here: the incidence of seed predators in tropical XSBN is lower in 2019 but becomes higher in 2020 as against temperate CBS. Violin plots show the distribution of measurements derived from each batch of seed samples (CBS: *n* = 7 and 13 in 2019 and 2020, respectively; XSBN: *n* = 26 and 27 in 2019 and 2020, respectively), with median and inter quartile range as dots and boxes. Results using seed predation rates as the metric and further including simulated data of *Pinus koraiensis* are similar and shown in Figure A1.

**FIGURE A1:**
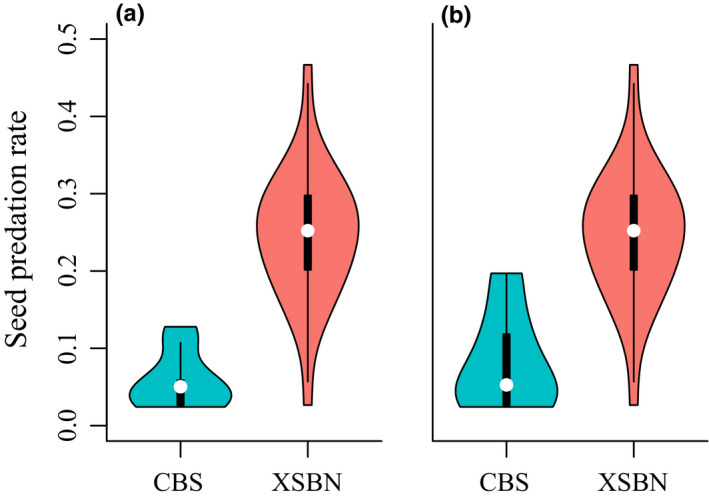
The tropical‐temperate comparisons of seed predation rate at the community‐wide level in 2020. The seed predation rate in tropical XSBN is higher than that in temperate CBS (a). The result remains similar when replacing (thus increasing) the seed predation rates of late September and early October batches in CBS with the simulated data of *Pinus koraiensis* (b). Violin plots show the distribution of measurements derived from each batch of seed samples (CBS: *n* = 13; XSBN: *n* = 27), with median and inter quartile range as dots and boxes. Results using the incidence of seed predators as the metric are similar and shown in Figure 2.

In community‐wide comparison, incidence of seed predator per batch was lower in the tropical XSBN (mean ± SE: 9.73 ± 1.72%) than in the temperate CBS (17.56 ± 1.07%) in 2019 (*F*_1,29_ = 22.58, *p* < .001; Figure 2a, Tables 1 and 2), contrary to latitudinal biotic interaction hypothesis. Opposite results were found in 2020, tropical incidence of seed predators (6.78 ± 0.86%) was higher than temperate counterparts (1.84 ± 0.43%; *F*_1,36_ = 19.44, *p* < .001; Figure 2b, Tables 1 and 2), supporting latitudinal biotic interaction hypothesis. Similar results were obtained using seed predation rate as the metric (Figure A1a, Tables 1 and 2). Similar results were obtained when we replaced (thus increased) the seed predation rates of late September and early October batches in CBS in 2020 with the simulated data of *Pinus koraiensis* (i.e., 19.7%, Figure A1b).

**TABLE 2:** Effect of study sites (XSBN vs. CBS) and sampling date (of each batch) on seed predation intensity at the community‐wide level

Year	Metric	Variable	*df*	*F*	*p*
2019	Incidence of seed predator	Site	1	22.577	**<.001**
		Date	1	0.463	.501
2020	Incidence of seed predator	Site	1	19.440	**<.001**
		Date	1	9.871	**.003**
	Seed predation rate	Site	1	85.874	**<.001**
			[1]	[43.398]	**[<.001]**
		Date	1	0.853	.362
			[1]	[0.460]	[.502]

*Note*: Linear models were conducted with the errors allowed to be correlated and/or have unequal variances to address temporal autocorrelation and heterogeneity of variance. Results incorporating simulated data of *Pinus koraiensis* are shown in square brackets. The *p* values <.05 are bolded.

**FIGURE 3:**
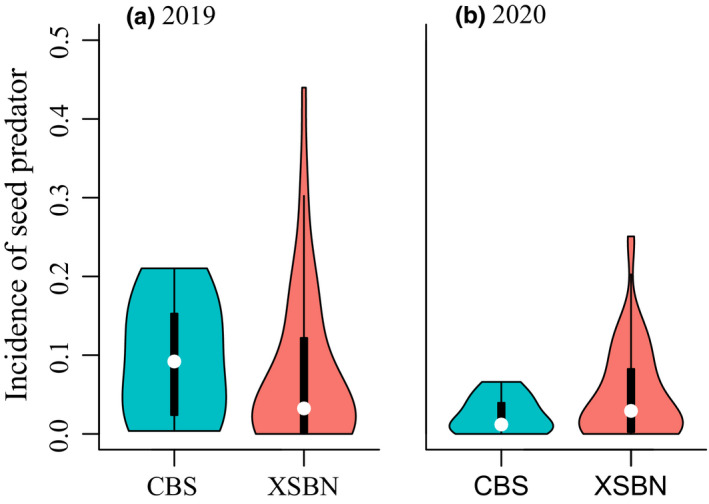
The tropical–temperate comparisons of the incidence of seed predators at the cross‐species level in (a) 2019 and (b) 2020. Different patterns are shown here: the incidence of seed predator in tropical XSBN is lower in 2019 but becomes statistically similar in 2020 as against temperate CBS. Violin plots show the distribution of measurements derived from each plant species with ≥50 seeds (CBS: *n* = 7 and 9 in 2019 and 2020, respectively; XSBN: *n* = 47 and 56 in 2019 and 2020, respectively), with median and inter quartile range as dots and boxes. Results using seed predation rate as the metric or two metrics unweighted by seed mass are similar and shown in Figures A2 and A3. Results remain similar when including simulated seed predation rate of *Pinus koraiensis* in CBS, as shown in Figure A4.

**FIGURE A2:**
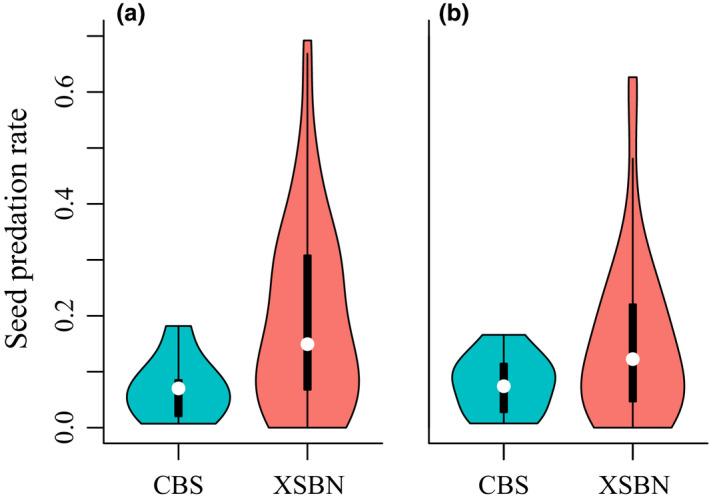
The tropical‐temperate comparisons of seed predation rate at the cross‐species level in 2020. Tropical XSBN is similar in seed predator rate to temperate CBS, regardless of the metric being weighted by seed mass (a) or not (b). Violin plots show the distribution of measurements derived from each plant species with ≥50 seeds (CBS: *n* = 9; XSBN: *n* = 56), with median and inter quartile range as dots and boxes. Results using the incidence of seed predators as the metric are similar and shown in Figures  3 and A3.

**FIGURE A3:**
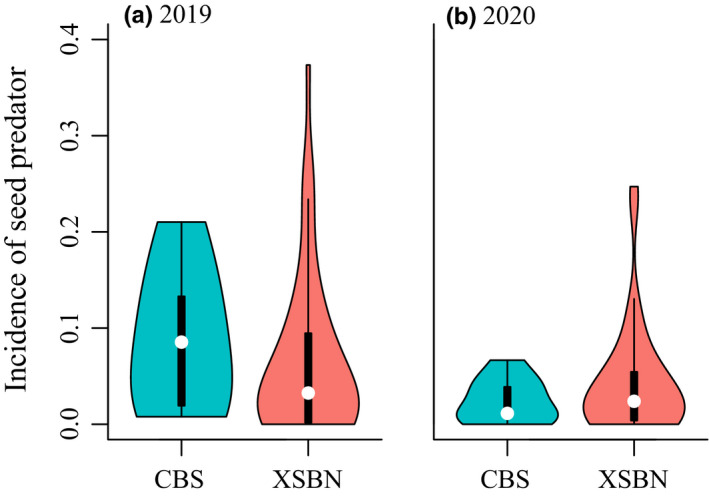
The tropical‐temperate comparisons of the incidence of seed predators *unweighted by seed mass* at the cross‐species level in (a) 2019 and (b) 2020. Different patterns are shown here: the unweighted metric in tropical XSBN is lower in 2019 but becomes statistically similar in 2020 as against temperate CBS. Violin plots show the distribution of measurements derived from each plant species with ≥50 seeds (CBS: *n* = 7 and 9 in 2019 and 2020, respectively; XSBN: *n* = 47 and 56 in 2019 and 2020, respectively), with median and inter quartile range as dots and boxes. Results using the weighted metric are similar and shown in Figure 3.

**FIGURE A4:**
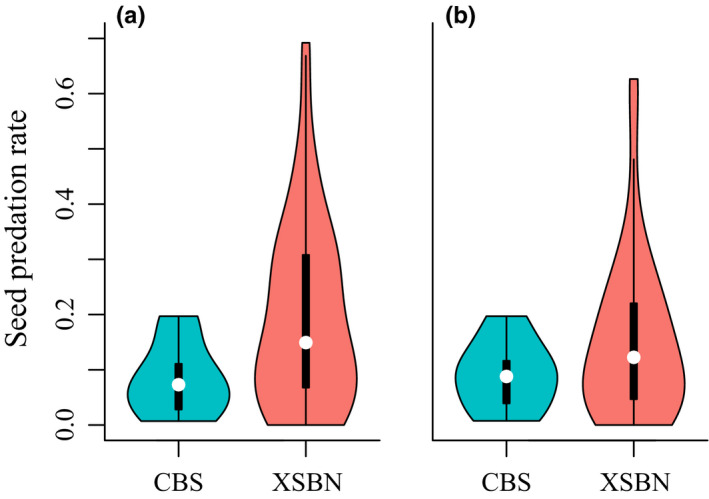
The tropical‐temperate comparisons of seed predation rate at the cross‐species level in 2020, including simulated data of *Pinus koraiensis* in CBS. Tropical XSBN is similar in seed predator rate to temperate CBS, regardless of the metric being weighted by seed mass (a) or not (b). Violin plots show the distribution of measurements derived from each plant species with ≥50 seeds (CBS: *n* = 10; XSBN: *n* = 56), with median and inter quartile range as dots and boxes. Results using real data are similar and shown in Figures 3, A2, and A3.

In cross‐species comparison, seven species in CBS and 47 species in XSBN with ≥50 seeds were retained in 2019, and nine species in CBS and 56 species in XSBN with ≥50 seeds were retained in 2020. The incidence of seed predator was lower in the tropical XSBN (7.64 ± 1.46%) than in the temperate CBS (9.42 ± 3.03%) in 2019 (*F*_1,50_ = 4.73, *p* = .03; Figure 3a, Tables 1 and 3), contrary to latitudinal biotic interaction hypothesis. The significant difference became missing in 2020 (tropical XSBN vs. temperate CBS: 5.11 ± 0.79% vs. 2.42 ± 0.79%, *F*_1,61_ = 0.40, *p* > .05; Figure 3b, Tables 1 and 3). Similar results were obtained using seed predation rate as the metric for the 2020 sample (Figure A2, Tables 1 and 3) or unweighted metric by seed mass (Figure A3, Tables 1 and 3). Similar results were obtained when we included the simulated data of *Pinus koraiensis* in 2020 (Figure A4, Tables 1 and 3).

**TABLE 3:** Effect of study sites (XSBN vs. CBS), fruit type (fleshy vs. dry fruit), and average seed mass (of each plant species) on seed predation intensity at the cross‐species level

Year	Metric	Variable	*df*	*F*	*p*	*λ*
2019	Incidence of seed predators	Site	1	4.725	**.034**	0.87
		Fruit type	1	0.024	.877	
		Seed mass	1	0.020	.889	
2020	Incidence of seed predators	Site	1	0.404	.527	0.49
		Fruit type	1	0.675	.414	
		Seed mass	1	6.508	**.013**	
	Seed predation rate	Site	1	2.651	.109	0.64 [0.80]
			1	[2.638]	[.109]	
		Fruit type	1	1.089	.301	
			1	[1.080]	[.303]	
		Seed mass	1	7.120	**.010**	
			1	[7.164]	**[.010]**	

*Note*: The phylogenetic generalized linear models were conducted to control plant phylogenetic relatedness. λ was used to measure phylogenetic signal in seed predation intensity, with λ = 0 indicating no phylogenetic signal and λ = 1 indicating a strong phylogenetic signal. Results incorporating simulated data of *Pinus koraiensis* are shown in square brackets. The *p* values <.05 are bolded. The results were similar when ignoring intraspecific variations in the seed mass and using unweighted metrics, as shown in Table A2.

## 4 DISCUSSION

Our study has demonstrated that tropical–temperate comparisons in insect seed predation could be higher, lower, or similar at the community‐wide (Figures 2 and A1, Tables 1 and 2) or cross‐species level (Figures 3 and A2–A4, Tables 1 and 3). We confirm the patterns by two metrics of seed predation intensity in 2020 (i.e., seed predation rate and incidence of seed predators) and by including simulated data of *Pinus koraiensis*.

Our results show that tropical forests do not consistently suffer greater losses to seed predators than temperate forests, and the study levels and years influence the latitudinal comparisons. The complex latitudinal patterns thus challenge the generality of the biotic interaction hypothesis, aligning with recent community‐wide surveys on granivory, herbivory, and predation on insects (Chen et al., 2017; Mottl et al., 2020; Zvereva, Zverev, Usoltsev, & Kozlov, 2020b).

### 4.1 Study levels

Our findings suggest that the latitudinal pattern of seed predation by insects at the community‐wide level does not necessarily match that averaged across individual plant species. The cross‐species level analyses ignore differences among species in seed production and weaken (in 2019, Table 1, Figures 2a vs. 3a) or even lose (in 2020, Table 1, Figures 2b vs. 3b) the latitudinal trend detected by community‐wide comparisons. The discrepancy between study levels was also demonstrated in insect folivory. Although herbivory values averaged across seven plant species decreased with latitude, community‐wide herbivory showed no statistically significant latitudinal pattern (Zvereva, Zverev, Usoltsev, & Kozlov, 2020b). It is thus not reasonable to assume data on the relative losses of seeds to insects collected from single species or averaged across multispecies can be used as a proxy for community‐wide seed losses in studies addressing spatial patterns of granivory.

Species‐ and community‐based approaches represent two interrelated but different questions in testing the latitudinal biotic interaction hypothesis (Anstett et al., 2016). Although both approaches face drawbacks, community‐wide studies are crucial for assessing the contribution of insects to community‐level (species composition and richness regulation) and ecosystem‐level processes (carbon and nutrient cycling; Zvereva, Zverev, Usoltsev, & Kozlov, 2020b), while species‐level studies are especially favorable in linking insect damage with plant defensive and nutritional traits (e.g., Moreira et al., 2018, 2021), and in answering the adaptive consequences of species interaction (e.g., Freeman et al., 2020).

### 4.2 Annual variations

Our findings further suggest that tropical vs. temperate comparisons existed between‐year variations at both study levels (Figures 2 and A1, Figures 3 and A2–A4). We propose the interplay between mast seeding and insect seed predator in temperate CBS as one of the causes. Although mast seeding occurred in both of our study sites (Dou et al., 2018; Qian et al., 2019), it was more pronounced in the temperate CBS, as indicated by a sharp increase of annual seed mass by three times (Table 1). According to the seed predator satiation hypothesis (Janzen, 1971; Kelly, 1994; Silvertown, 1980), plants suffer less seed predation in high‐seed years than in low‐seed years. The insect predators are easier to satiate where one species dominates local seed production and fewer alternate resources are available, so masting is especially well‐developed in temperate forests than in biodiverse tropical forests (Kelly & Sork, 2002; Pearse et al., 2020; Zwolak et al., 2022). Consequently, the seed production and associated seed predation intensity of individual dominant species are more likely to be reflected in the community‐wide measurements at higher latitudes.

In this study, the mast seeding of dominant tree species in CBS was so overwhelming that it largely determined community‐wide seed predation intensity well below or above tropical measurements. For example, mast seeding of *Tilia amurensis* in 2020 decreased CBS's community‐wide seed predator incidence per batch from 4.10 ± 0.78% (excluding *T. amurensis*) to 1.84 ± 0.43% (vs. 6.78 ± 0.86% in XSBN, Table 1), whereas mast seeding of *Fraxinus mandshurica* in 2019 increased CBS's community‐wide seed predator incidence per batch from 10.77 ± 3.29% (excluding *F. mandshurica*) to 17.56 ± 1.07% (vs. 9.73 ± 1.72% in XSBN, Table 1). It is worth noting that the masting seeding, as a population‐level adaptive reproductive strategy (Kelly & Sork, 2002), does not necessarily lower seed predation intensity at the community‐wide level.

The predator satiation effect of masting is complicated by the intricacies of predator life histories (Zwolak et al., 2022). The life history, mobility, and diet breadth of insect predators and their interactions with other seed predators might shape seed predation patterns and masting dynamics (Bogdziewicz et al., 2021; Kelly, 2021). However, the insect seed predator guild is poorly studied in our study sites. Further research on insect predators' taxonomies, host specificity, and life histories is needed to comprehend the causes and selective consequences of mast seeding in our study sites.

The annual variations in tropical–temperate comparison (Figures 2 and 3) also indicate that measuring a snapshot of granivory at the community‐wide or cross‐species level in a specific year might provide opposite and misleading patterns. The among‐year variations also exist in leaf herbivory (Adams & Zhang, 2009; Zhang et al., 2011; Zvereva, Zverev, & Kozlov, 2020a). A notable example is insect outbreaks, which are common from a phytocentric perspective and have major top‐down effects on plant communities and ecosystems (Carson et al., 2008). We argue the annual variations on some occasions could determine the latitudinal pattern present or absent (Zvereva, Zverev, & Kozlov, 2020a) and change the direction to be positive or negative. Therefore, temporal variation should be considered in testing the biotic interaction hypothesis, and long‐term comparisons are recommended (Anstett et al., 2016).

### 4.3 Interspecific variations

The interspecific variations in seed predation intensity are ubiquitous in the community (Gripenberg, 2018; Jeffs et al., 2018), and understanding the causes is central to studying plant–enemy interaction (Gripenberg et al., 2019). A variety of plant traits and phylogeny have been proposed to influence plant species' susceptibility to predispersal seed predators (Gripenberg et al., 2019). However, neither has received general support at large spatial scales (Basset et al., 2018; Chen & Moles, 2018; Moles & Westoby, 2003), indicating they operate in complex manners or at a fine spatial scale. Our study confirmed such inconsistency and showed phylogenetic signals were moderate to strong (*λ* = 0.49–0.87, Table 3), seed mass was influential in one study year (i.e., 2020), and fruit types were negligible. The patterns of seed predation intensity across biomes are also likely to be driven primarily by factors not investigated here (such as density‐dependent effects mentioned above), and specific research incorporating comprehensive influential factors is further needed.

We statistically accounted for phylogenetic relatedness and two plant traits in the datasets to reduce the variation associated with tropical and temperate plant assemblages. An alternative approach recommended is choosing a subset of plant species from the tropical community that phylogenetically matches the temperate counterpart (Anstett et al., 2016). However, a phylogenetic match does not mean a functional match, as plant functional traits (e.g., seed mass) were not readily incorporated. Seed mass is a central trait in seed functional ecology and is associated with seed defense and nutrition (Fenner & Thompson, 2005). Seed mass also exhibits a striking negative latitudinal pattern, declining by 2–3 orders of magnitude between the equator and 60° (Moles et al., 2007; Moles & Westoby, 2003); thus, it is not feasible to choose a subset of plant species with comparable seed mass with temperate counterpart. Moreover, phylogenetically selected species usually account for a tiny proportion of plant assemblage in the biodiverse tropical forest, making representativeness insufficient.

## 5 CONCLUSION

We applied a consistent community‐wide sampling design in a pair of tropical and temperate forests in mainland East Asia to study predispersal seed predation by insects. Our results reveal every latitudinal pattern associated with different study levels and years, to some extent reflecting the controversy surrounding the biotic interaction hypothesis in a single study. The latitudinal pattern of species interaction could be far more complicated due to interspecific, temporal, and other variations (Anstett et al., 2014; Coley & Barone, 1996). Like in testing the density‐dependent effects (Bogdziewicz et al., 2021; Cannon et al., 2021), incorporating and addressing high and pervasive variation in natural systems is vital in developing a realistic assessment of the latitudinal biotic interaction hypothesis.

## AUTHOR CONTRIBUTIONS

**Wenlan Wu:** Data curation (Equal); Formal analysis (Equal); Investigation (Equal); Methodology (Equal); Writing—original draft (Lead); Writing—review and editing (Equal). **Xiaoxue Wang:** Data curation (Equal); Formal analysis (Equal); Investigation (Equal); Methodology (Equal); Writing—original draft (Supporting); Writing—review and editing (Equal). **Tao Zhao:** Data curation (Equal); Formal analysis (Supporting); Writing—review and editing (Equal). **Wenfu Zhang:** Data curation (Equal); Investigation (Lead); Project administration (Supporting); Resources (Equal); Writing—review and editing (Equal). **Shuai Fang:** Data curation (Equal); Investigation (Lead); Project administration (Supporting); Resources (Equal); Writing—review and editing (Equal). **Yu Xu:** Conceptualization (Supporting); Formal analysis (Supporting); Funding acquisition (Supporting); Methodology (Equal); Writing—review and editing (Equal). **Kai Zhang:** Conceptualization (Lead); Data curation (Equal); Formal analysis (Equal); Funding acquisition (Lead); Methodology (Lead); Project administration (Lead); Supervision (Lead); Writing—review and editing (Lead).

## CONFLICT OF INTEREST

None declared.

## Data Availability

The data and R script are openly available in Dryad DOI: 10.5061/dryad.9p8cz8wkf.

## APPENDIX A

**TABLE A1:** Seed predation intensity (per species) *unweighted by seed mass* in two study sites in 2019–2020

Site	2019	2020	
	Incidence of seed predators	Seed predation rate	Incidence of seed predators
Tropical XSBN	6.40 ± 1.22%	15.22 ± 1.96%	4.26 ± 0.76%
Temperate CBS	8.68 ± 2.92%	7.83 ± 1.74%	2.43 ± 0.77%
		[9.02 ± 1.95%]	

*Note*: Mean ± SE values are presented. Measurements incorporating simulated data of *Pinus koraiensis* are shown in square brackets. Weighted measurements, which incorporated the intraspecific variations in seed mass, are shown in Table 1.

**TABLE A2:** Effect of study sites (XSBN vs. CBS), fruit type (fleshy vs. dry fruit), and average seed mass (of each plant species) on seed predation intensity *unweighted by seed mass* at the cross‐species level

Year	Metric	Variable	*df*	*F*	*p*	λ
2019	Incidence of seed predators	Site	1	4.759	**.034**	.83
		Fruit type	1	0.289	.594	
		Seed mass	1	0.300	.586	
2020	Incidence of seed predators	Site	1	0.118	.732	.55
		Fruit type	1	0.636	.428	
		Seed mass	1	7.400	**.008**	
	Seed predation rate	Site	1	1.045	.311	.66 [.82]
			1	[1.037]	[.313]	
		Fruit type	1	1.463	.231	
			1	[1.461]	[.231]	
		Seed mass	1	10.079	**.002**	
			1	[10.119]	**[.002]**	

*Note*: The phylogenetic generalized linear models were conducted to control plant phylogenetic relatedness. λ was used to measure phylogenetic signal in seed predation intensity, with λ = 0 indicating no phylogenetic signal and λ = 1 indicating a strong phylogenetic signal. Results incorporating simulated data of *Pinus koraiensis* are shown in square brackets. The *p* values < .05 are bolded. Results with weighted seed predation intensity, which incorporated the intraspecific variations in seed mass, are shown in Table 3.
